# Efficacy and Safety of a Telemedicine System in Patients With Gestational Diabetes Mellitus (TELEGLAM): Single-Center, 2-Arm, Randomized, Open-Label, Parallel-Group Study

**DOI:** 10.2196/72242

**Published:** 2026-05-08

**Authors:** Kazuki Aoyama, Yuya Nakajima, Shu Meguro, Yasunori Sato, Rei Goto, Mariko Hida, Takeshi Arimitsu, Yoshifumi Kasuga, Mamoru Tanaka, Hiroshi Itoh, Kaori Hayashi

**Affiliations:** 1 Department of Endocrinology, Metabolism and Nephrology, Keio University School of Medicine, 35 Shinanomachi, Shinjuku-ku, Tokyo, 160-0016, Japan, 81 3-5363-3797; 2 Center for Preventive Medicine, Keio University School of Medicine, Tokyo, Japan; 3 Department of Biostatistics, Keio University School of Medicine, Tokyo, Japan; 4 Graduate School of Business Administration, Keio University,, Kanagawa, Japan; 5 Department of Pediatrics, Keio University School of Medicine, Tokyo, Japan; 6 Department of Obstetrics and Gynecology, Keio University School of Medicine, Tokyo, Japan

**Keywords:** gestational diabetes mellitus, telemedicine, mobile health, patient satisfaction, costs

## Abstract

**Background:**

In the management of gestational diabetes mellitus (GDM), the usual medical treatment requires frequent visits for glucose monitoring and insulin dose adjustment, and this imposes significant physical, psychological, and economic burdens on pregnant women. As mobile health platforms become increasingly integrated into diabetes care, telemedicine may help alleviate these burdens; however, evidence evaluating its effectiveness as a replacement for routine in-person GDM care remains limited.

**Objective:**

This study aims to evaluate the impact of telemedicine on the quality of life and costs for patients with GDM requiring insulin therapy.

**Methods:**

This single-center, 2-arm, randomized, open-label, parallel-group study included patients with GDM who started insulin injection therapy. Participants were randomized to either the telemedicine or standard face-to-face care groups for 10 (SD 2) weeks. The telemedicine intervention used a smartphone-linked platform that enabled the automatic transfer of glucose data from connected glucose meters and facilitated real-time video consultations. Primary end points included costs and patient satisfaction. Costs were assessed using claims data, transportation calculations, and wage-based productivity losses, while patient satisfaction was evaluated through changes in the Problem Areas in Diabetes Survey and Diabetes Therapy-Related Quality of Life questionnaire scores. Secondary outcomes included glycemic control and perinatal outcomes.

**Results:**

In total, 38 participants were included, with 18 assigned to the telemedicine group and 20 to the standard care group. Total costs (32,712, 95% CI 15,412‐50,013 vs 59,202, 95% CI 42,603‐75,800 Japanese yen; $284, 95% CI 134‐435 vs $515, 95% CI 370‐659, purchasing power parity [PPP]–adjusted; *P*=.01), direct non–health care costs (922, 95% CI −240 to 2084 vs 2561, 95% CI 1447‐3676 yen; $8, 95% CI −2 to 18 vs $22, 95% CI 13 to 32 PPP-adjusted; *P*=.02), and indirect costs (8981, 95% CI −7119 to 25,082 vs 32,832, 95% CI 17,384‐48,279 yen; $78, 95% CI −62 to 218 vs $285, 95% CI 151‐420 PPP-adjusted; *P*=.01) reduced significantly in the telemedicine group compared with the standard care group. The improvements in the Problem Areas in Diabetes Survey (−7.6, 95% CI −13.7 to −1.4; *P*=.02) and Diabetes Therapy–Related Quality of Life domain 1 (10.5, 95% CI 0.9-20.1; *P*=.03) scores from the baseline were significantly greater in the telemedicine group than that in the standard care group. Nonetheless, glycemic control and frequency of perinatal complications were comparable between the 2 groups. Consultation time was similar across groups, suggesting no added workload for clinicians.

**Conclusions:**

In this randomized trial, mobile health–enabled telemedicine safely replaced routine in-person visits for patients with GDM requiring insulin therapy. Telemedicine significantly reduced psychological and economic burdens without compromising glycemic or perinatal outcomes, demonstrating its value as a patient-centered and cost-efficient model of care. These findings support the broader implementation of mobile-based telemedicine approaches in GDM management.

## Introduction

### Background

The prevalence of gestational diabetes mellitus (GDM) is rising globally due to increasing obesity rates and an aging population of women of childbearing age. GDM affects approximately 6% to 15% of all pregnancies [1] and increases the risk of adverse perinatal outcomes, such as cesarean section, large-for-gestational-age (LGA) infants, macrosomia, shoulder dystocia, and neonatal hypoglycemia [2]. Additionally, GDM raises the long-term complication risks of obesity and impaired glucose metabolism in mothers and infants [3]. Therefore, strict blood glucose management through diet, exercise, and pharmacotherapy is essential. However, conventional treatment requires frequent visits every 2 to 3 weeks for glucose monitoring, thereby imposing significant physical, psychological, and economic burdens on pregnant women. Systematic reviews have revealed that these patients experience a significant decrease in the quality of life (QOL) [4]. Furthermore, evidence from Italy indicates that health care expenditures are 29.2% higher in pregnancies complicated by GDM than in those without GDM [5].

### Rationale and Knowledge Gap

Recently, telemedicine, defined as the delivery of health care and the exchange of health care information across distances [6], has become widely prevalent. As a core component of mobile health (mHealth), smartphone-based telemedicine platforms enable real-time data sharing and remote clinical support. The effectiveness and economic efficiency of telemedicine have been demonstrated for many diseases [7-9]. Thus, further research into the safety and effectiveness of this approach for patients with GDM, and its impact on psychological and socioeconomic burdens is required.

Previous studies have shown significant findings. A meta-analysis of 7 randomized controlled trials (RCTs) demonstrated that telemedicine significantly improved Hemoglobin A_1c_ (HbA_1c_) levels in patients with GDM [10]. A subsequent meta-analysis of 32 RCTs reported a reduced risk of perinatal complications using telemedicine [11]. However, studies on patient satisfaction with telemedicine and its impact on costs among this population remain limited. Mackillop et al [12] conducted an RCT comprising 203 patients with GDM, investigating the effectiveness of a mobile phone–based real-time blood glucose management system and assessing patient satisfaction and direct health care costs as secondary outcomes. The satisfaction score, assessed using the Maternity Diabetes Treatment Satisfaction Questionnaire, was slightly higher in the intervention group. However, the direct health care costs did not differ significantly between the 2 groups, and insulin users, who were considered to have a particularly high level of psychological burden, were excluded. Furthermore, in a nonrandomized controlled trial involving 161 patients with GDM, direct health care costs were evaluated between a conventional therapy group and a group receiving additional support through the online analysis of capillary glucose data. The intervention group showed a 16% reduction in direct health care costs. However, costs other than direct health care costs were not evaluated in this study [13].

### Objective of the Study

Thus, based on a review of previous studies, the safety of telemedicine for patients with GDM is becoming clearer. However, no studies in Japan or other countries have examined the effects of telemedicine on psychological burden or costs as primary outcomes for patients with GDM. Furthermore, no reports have revealed how telemedicine use in this population affects not only direct health care costs but also direct non–health care costs, such as transportation expenses or indirect costs, such as production loss due to absenteeism. Therefore, we conducted a prospective randomized controlled trial to investigate the impact of telemedicine on QOL and costs, particularly in patients with GDM who received insulin injections.

## Methods

### Study Design and Participants

TELEGLAM (efficacy and safety of a telemedicine system in patients with gestational diabetes mellitus) was a single-center, 2-arm, randomized, open-label, parallel-group study. We included patients attending the Department of Endocrinology, Metabolism and Nephrology, Keio University School of Medicine in Japan. The study was performed between February 7, 2022, and March 3, 2024. Its protocol has been previously described in detail [14]. Participants were eligible for inclusion if they met the following criteria: (1) diagnosed with GDM through an oral glucose tolerance test (OGTT) by 29 weeks and 6 days of gestation, underwent self-monitoring of blood glucose, and received insulin injections; (2) provided written informed consent; and (3) could operate the telemedicine system. In our hospital, the first trimester screening for GDM was performed for high-risk patients as reported previously [15]. If the first trimester screening for GDM was negative or positive but OGTT was negative, pregnant women had to undergo the 50-g glucose challenge test at approximately 24 gestational weeks. If the patients were glucose challenge test–positive, they underwent OGTT at 24 to 28 gestational weeks [16]. Furthermore, GDM was diagnosed following the International Association of Diabetes and Pregnancy Study Groups Consensus Panel [17]: fasting plasma glucose ≥5.1 mmol/L (92 mg/dL) and/or 1-hour plasma glucose ≥10.0 mmol/L (180 mg/dL), and/or 2-hour plasma glucose ≥8.5 mmol/L (153 mg/dL). The target range of glycemic control was set at a preprandial blood glucose level of ˂5.6 mmol/L (100 mg/dL) and a 2-hour postprandial blood glucose level of ˂6.7 mmol/L (120 mg/dL). Insulin therapy was initiated if these targets were not achieved through diet and exercise. Exclusion criteria included (1) a GDM diagnosis after 30 weeks of gestation, (2) overt diabetes in pregnancy, (3) type 2 diabetes, (4) type 1 diabetes, (5) serious uncontrolled complications, (6) pacemakers or other implantable medical devices, (7) unwillingness to participate in the study, (8) being judged inappropriate to participate in the study by the physicians (eg, difficulties in understanding Japanese, refusal to treat GDM, difficulty in making regular visits to the hospital), (9) those without the necessary internet access for telemedicine, and (10) those who did not use smartphones.

### Ethical Considerations

The study protocol was approved by the Keio University School of Medicine Ethics Committee on February 7, 2022 (approval number: 20211125) and registered with the University Hospital Medical Information Network Clinical Trials Registry (UMIN000047009) in Japan. The protocols adhered to the principles of the Declaration of Helsinki. Written informed consent was obtained from all participants prior to enrollment. Participants were informed of the study purpose, procedures, potential risks, and their right to withdraw at any time without providing a reason. All collected data were deidentified before analysis. Clinical information and glucose data were stored on secure institutional servers accessible only to authorized study personnel. No personally identifiable information was used in the analytic dataset. Participants did not receive financial or material compensation for their participation in this study.

### Intervention and Control

**Figure 1.**
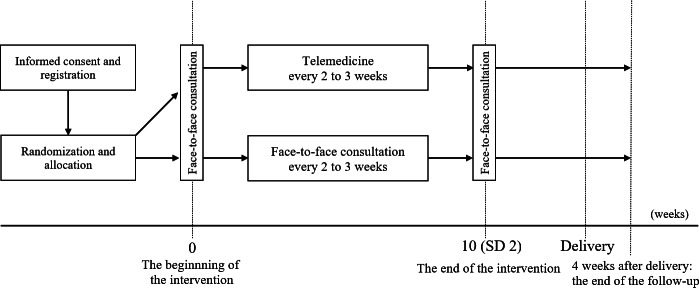
Trial design. In the intervention group, face-to-face consultations were conducted at the beginning and end of the intervention period, and telemedicine was conducted every 2 to 3 weeks in the interim period. In the control group, the patients were examined face-to-face every 2 to 3 weeks, as usual.

Telemedicine was provided to the patients in the intervention group using the MeDaCa telemedicine system developed by Medical Data Card Inc (Tokyo, Japan). This system connects a medical facility’s computer with a patient’s smartphone, tablet, or computer via the internet, enabling real-time video consultations similar to face-to-face visits. We did not include lifestyle interventions using smartphone apps, which enabled us to examine the effects of switching from face-to-face consultations to telemedicine. At Keio University, the One Touch Verio Reflect glucose meter (LifeScan Inc) was linked to MeDaCa. This allowed MeDaCa to automatically import blood glucose measurement data. The intervention period began at the first outpatient visit after the initiation of insulin injection and self-monitoring of blood glucose and ended 10 (SD 2) weeks later (Figure 1). In the intervention group, face-to-face consultations were conducted at the beginning and end of the intervention period, and telemedicine was conducted every 2 to 3 weeks in the interim period. In the control group, the patients were examined face-to-face every 2 to 3 weeks as usual. During this study, unscheduled visits were available at any time in both groups. Nurses provided telephone support as needed, and guidance on diet and exercise was similarly provided by doctors, nurses, and registered dietitians in both groups. The prenatal checkup was conducted as usual. Baseline and follow-up clinical information was obtained from outpatient visits and the hospital’s electronic medical record system, and glucose data were transmitted through the telemedicine system. No automated or manual reminders were used during the intervention.

### Outcomes

#### Primary Outcomes

In this study, we performed a cost-consequence analysis (CCA). CCA is defined as “a form of health economic evaluation study in which all direct and indirect costs and a catalog of different outcomes of all alternatives are listed separately” [18]. Therefore, multiple primary end points were established, focusing on health economic indicators and patient satisfaction. The health economic indicators included (1) direct health care costs, (2) direct non–health care costs, and (3) indirect costs. The actual costs incurred by medical examinations were evaluated as direct health care costs using the claims data, while direct non–health care costs, such as transportation costs, were calculated using Google Maps based on the patient’s home or work address. Indirect costs, defined as production losses due to the inability to work because of the disease, were calculated from sick leave hours and the average wage in Japan if the patient was employed. Additionally, indirect costs associated with childcare were also considered, such as leaving a child with a babysitter for a hospital visit or a spouse taking a leave. The costs are presented in Japanese yen (¥), and US $1 was equivalent to 140.56 yen based on the exchange rate between January 2023 and January 2024. Furthermore, to facilitate international cost comparisons, we also converted all cost variables into purchasing power parity (PPP)–adjusted international dollars using a conversion factor of 115 yen per international dollar.

Moreover, we assessed patient satisfaction using the Problem Areas in Diabetes Survey (PAID) [19,20] and the Diabetes Therapy–Related QOL (DTR-QOL) questionnaire [21], both of which were used in their original, unmodified forms. Notably, PAID is a self-administered questionnaire comprising 20 items that measure diabetes-related psychological burdens. Each question was rated on a scale of 1 to 5, and the total score was evaluated. A lower score indicated a lesser psychological burden. The DTR-QOL is a questionnaire that assesses the influence of diabetes treatment on patient QOL, regardless of the treatment method. It comprises 29 questions divided into 4 domains, with each question answered on a scale of 1 to 7. Domain 1 assesses the burden on social and daily activities, domain 2 evaluates anxiety and dissatisfaction with treatment, domain 3 assesses hypoglycemia, and domain 4 evaluates satisfaction with the treatment. A higher score indicates a better QOL. The results were presented as the total scores for each domain, converted to a scale of 100 points. Participants completed the questionnaires at both the start and the end of the intervention, and the change from baseline was evaluated.

#### Secondary Outcomes

Secondary outcomes included changes in glycemic control indicators and metabolic parameters between the start and end of the intervention and perinatal outcomes. We evaluated fasting and postprandial blood glucose levels from self-monitoring records, total insulin dose, plasma glucose, HbA_1c_, and glycated albumin as glycemic control indicators. Furthermore, maternal weight, BMI, systolic blood pressure (SBP), diastolic blood pressure (DBP), low-density lipoprotein cholesterol, high-density lipoprotein cholesterol, triglyceride, C peptide, and the urinary albumin-to-creatinine ratio were evaluated as metabolic parameters. BMI was defined as weight in kilograms divided by the square of height in meters. Perinatal outcomes were evaluated based on the following: gestational age; mode of delivery; obstetric complications occurring during the perinatal period, such as premature rupture of the membrane, postpartum hemorrhage, and shoulder dystocia; maternal complications such as gestational hypertension; fetal indices including sex, height, weight, neonatal hypoglycemia, Apgar score (1 min/5 min), umbilical artery pH, macrosomia (>4000 g), low birth weight (<2500 g), LGA (>90th percentile), and small for gestational age (<10th percentile); incidence of congenital diseases; and admission to the neonatal intensive care unit. Additionally, neonatal hypoglycemia was defined as a blood glucose level of <40 mg/dL, and blood glucose was measured using point-of-care testing equipment. We used an intervention criterion of <50 mg/dL.

As an exploratory evaluation item, we measured the time spent in consultation based on the medical records for both groups.

### Sample Size

The exploratory nature of the study made it difficult to predict parameter changes because no clinical study had used the PAID or the DTR-QOL for patients with GDM. Of the primary end points, the PAID was assumed to be the least likely to change with intervention. We estimated PAID scores to be similar between the groups before the intervention, with a 10-point difference emerging postintervention based on previous studies using the PAID [22,23]. To achieve a significance level of .05 in a 2-sided test with 80% power, assuming an SD of 10 points, the required sample size was estimated at 32 participants. Accounting for a 20% dropout rate, the target sample size was set at 40 participants.

### Randomization

We performed randomization in a 1:1 ratio using a modified minimization method with a biased-coin assignment balancing maternal age (≤39 years old vs ≥40 years old), prepregnancy obesity, history of GDM, number of fetuses (singleton vs twins or higher order pregnancy), and ethnicity. The University Hospital Medical Information Network Internet Data and Information System for Clinical and Epidemiological Research (Cloud version) was used for randomization. A physician not involved in the study created the allocation registration form. Participants and physicians were not blinded to the treatment allocation.

### Statistical Analysis

Primary and secondary outcomes were analyzed in the full analysis set, which included all patients who completed at least 2 online consultations during the study period and did not violate any of its protocols. Summary statistics comprised frequencies and proportions for categorical variables and means and SDs or medians with IQRs for continuous variables for the baseline characteristics. Categorical variables were compared using chi-square tests, while continuous variables were compared using the 2-tailed *t* test or Wilcoxon rank-sum test. For the primary end points, representative statistics were calculated for each measurement point, and an analysis of covariance was conducted. The analysis of covariance model included the following covariates: treatment group, maternal age, prepregnancy obesity, history of GDM, and number of fetuses. Secondary end points were analyzed similarly to the primary end points. All comparisons were preplanned, and all *P* values were 2-sided. A *P* value of <.05 was considered statistically significant. Statistical analyses were conducted using IBM SPSS Statistics software for Windows (version 28.0.0.0; IBM Corp).

## Results

### Patient Flow and Study Population

**Figure 2.**
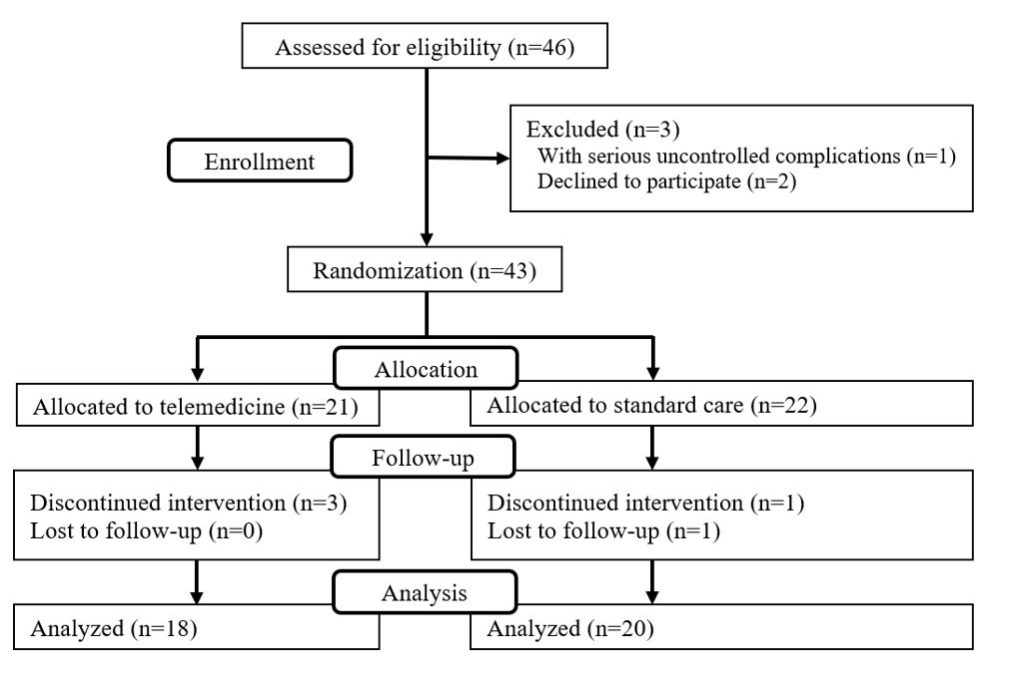
Flow diagram of patient enrollment.

In total, 46 patients were initially selected for the study between February 7, 2022, and March 3, 2024. Of these, 1 patient was excluded due to serious uncontrolled complications, and 2 patients declined to participate, resulting in the enrollment of 43 patients. We randomly assigned these patients to the telemedicine group (intervention group) or the standard care group (control group). In the telemedicine group, 3 patients delivered before the study concluded, whereas in the standard care group, 1 patient delivered early, and 1 patient was lost to follow-up. Consequently, the full analysis set included 38 patients: 18 in the telemedicine group and 20 in the standard care group (Figure 2).

### Baseline Characteristics

Baseline characteristics were comparable between the groups, excluding C peptide, triglyceride, and the proportion of target glucose levels in the previous 7 days (Table 1). The mean age was 37.1 (SD 4.6) years and 35.8 (SD 4.1) years in the intervention and control groups, respectively, with a BMI of 24.7 (SD 4.3) and 24.0 (SD 4.1) kg/m², respectively. All patients, except 1 in the control group, were Japanese. The proportion of working patients was 72% (n=13) and 70% (n=14) in the intervention and control groups, respectively. Additionally, the mean daily insulin dose at the start of the intervention was 10.5 (SD 8.4) U/day and 9.8 (SD 9.1) U/day, respectively. At baseline, there were no significant differences in the PAID and DTR-QOL questionnaire scores between the 2 groups.

**Table 1.** Baseline characteristics.

Characteristics	Telemedicine group (n=18)	Standard care group (n=20)
Maternal age (y), mean (SD)	37.1 (4.6)	35.8 (4.1)
Gestational age at diagnosis (wk), median (IQR)	19 (14-26)	18 (14-26)
Height (cm), mean (SD)	159.6 (6.4)	157.9 (4.8)
Body weight (kg), mean (SD)	62.9 (12.5)	60.0 (11.3)
BMI (kg/m^2^), mean (SD)	24.7 (4.3)	24.0 (4.1)
Systolic blood pressure (mm Hg), mean (SD)	109.1 (14.6)	110.1 (14.5)
Diastolic blood pressure (mm Hg), mean (SD)	66.3 (11.9)	65.1 (11.8)
Body weight before pregnancy (kg), mean (SD)	59.6 (12.7)	56.4 (11.3)
BMI before pregnancy (kg/m^2^), mean (SD)	23.4 (4.7)	22.6 (4.1)
Obesity before pregnancy, n (%)	6 (33)	8 (40)
Gravidity, median (IQR)	2 (1-2)	1.5 (1-2)
Parity, median (IQR)	0 (0-1)	0 (0-1)
Previous GDM^a^, n (%)	3 (17)	3 (15)
Family history of type 2 diabetes, n (%)	8 (44)	11 (55)
Nationality, n (%)		
Japanese	18 (100)	19 (95)
Chinese	0 (0)	1 (5)
Employed, n (%)	13 (72)	14 (70)
Number of fetuses, n (%)		
Singleton	18 (100)	20 (100)
Twins or higher	0 (0)	0 (0)
OGTT^b^ results (mg/dL), mean (SD)		
Fasting plasma glucose	90.6 (7.4)	92.0 (8.4)
1-hour plasma glucose	187.7 (21.4)	178.7 (30.8)
2-hour plasma glucose	152.8 (32.5)	158.0 (17.2)
Plasma glucose (mg/dL), mean (SD)	104.6 (21.0)	93.1 (23.2)
HbA_1c_^c^ (%), mean (SD)	5.3 (0.3)	5.3 (0.4)
HbA_1c_ (mmol/mol), mean (SD)	34.5 (2.8)	33.9 (4.0)
GA^d^ (%), mean (SD)	12.2 (1.2)	12.5 (1.1)
C peptide (ng/mL), mean (SD)	5.0 (3.2)	2.7 (1.8)
LDL-C^e^ (mg/dL), mean (SD)	123.6 (37.8)	126.6 (28.3)
HDL-C^f^ (mg/dL), mean (SD)	73.1 (16.8)	75.6 (11.6)
Triglyceride (mg/dL), mean (SD)	252.4 (92.2)	195.0 (79.1)
Urinary albumin-to-creatinine ratio (mg/gCr), mean (SD)	8.9 (7.6)	6.5 (3.3)
Total insulin dose (U/day), mean (SD)	10.5 (8.4)	9.8 (9.1)
Proportion of target glucose levels in the previous 7 days (%), mean (SD)	71.1 (17.4)	83.9 (11.0)
Questionnaire, mean (SD)		
PAID^g^	46.3 (14.6)	46.6 (12.6)
DTR-QOL1^h^	58.0 (20.0)	62.4 (19.8)
DTR-QOL2	63.4 (18.8)	60.5 (19.1)
DTR-QOL3	78.8 (24.5)	85.5 (19.0)
DTR-QOL4	47.8 (20.9)	50.7 (16.3)
DTR-QOL total	60.9 (13.6)	63.4 (13.3)

a GDM: gestational diabetes mellitus.

b OGTT: oral glucose tolerance test.

c HbA_1c_: hemoglobin A_1c_.

d GA: glycated albumin.

e LDL-C: low-density lipoprotein cholesterol.

f HDL-C: high-density lipoprotein cholesterol.

g PAID: Problem Areas in Diabetes Survey.

h DTR-QOL: Diabetes Therapy-Related QOL.

### Primary Outcomes

The results of the CCA are shown in Tables2  3. Regarding health economic indicators, the direct health care costs were comparable between the 2 groups. However, the telemedicine group had significantly lower direct non–health care costs (922, 95% CI −240 to 2083 vs 2561, 95% CI 1447‐3676 yen; $8 95% CI −2 to 18 vs $22, 95% CI 13‐32 PPP-adjusted; *P*=.02), indirect costs (8981, 95% CI −7119 to 25,082 vs 32,832, 95% CI 17,384‐48,279 yen; $78, 95% CI −62 to 218 vs $285, 95% CI 151‐420 PPP-adjusted; *P*=.01), and total costs (32,712, 95% CI 15,412‐50,013 vs 59,202, 95% CI 42,603‐75,800 yen; $284, 95% CI 134‐435 vs $515, 95% CI 370‐659 PPP-adjusted; *P*=.01) than the standard care group. Notably, policies were implemented during the COVID-19 pandemic to promote remote telemedicine to reduce crowding in medical institutions. However, as the number of infections decreased, these policies were discontinued, leading to changes in direct health care costs related to telemedicine during the study period. Therefore, we also analyzed the direct health care costs assuming telemedicine costs were equivalent to face-to-face consultations, and the results were consistent with those obtained using actual costs (Multimedia Appendix 1).

**Table 2.** Primary outcomes—cost consequence analysis of health economic indicators.

Variables	Telemedicine group (n=18)		Standard care group (n=20)		Mean difference		*P* value
	Yen (95% CI)	PPP^a^-adjusted dollars (95% CI)	Yen (95% CI)	PPP-adjusted dollars (95% CI)	Yen (95% CI)	PPP-adjusted dollars (95% CI)	
Costs							
Direct health care costs	22,809 (20,108 to 25,511)	198 (175 to 222)	23,809 (21,217 to 26,400)	207 (184 to 230)	−999 (−4103 to 2104)	−9 (−36 to 18)	.52
Direct non–health care costs	922 (−240 to 2084)	8 (−2 to 18)	2561 (1447 to 3676)	22 (13 to 32)	−1639 (−2974 to −304)	−14 (−26 to −3)	.02
Indirect costs	8981 (−7119 to 25,082)	78 (−62 to 218)	32,832 (17,384 to 48,279)	285 (151 to 420)	−23,851 (−42,349 to −5353)	−207 (−368 to −47)	.01
Total costs	32,712 (15,412 to 50,013)	284 (134 to 435)	59,202 (42,603 to 75,800)	515 (370 to 659)	−26,489 (−46,366 to −6613)	−230 (−403 to −58)	.01

a PPP: purchasing power parity.

**Table 3.** Primary outcomes—cost consequence analysis of patient satisfaction.

Variables	Telemedicine group (n=18)		Standard care group (n=20)		Difference in change (95% CI)	*P* value
	After intervention (95% CI)	Change from baseline (95% CI)	After intervention (95% CI)	Change from baseline (95% CI)		
Consequences						
PAID^a^	39.7 (31.9 to 47.4)	–3.8 (–9.1 to 1.6)	48.8 (41.3 to 56.2)	3.8 (–1.4 to 8.9)	–7.6 (–13.7 to 1.4)	.02
DTR-QOL^b^ domain 1	68.0 (55.7 to 80.2)	7.8 (–0.5 to 16.2)	60.9 (49.2 to 72.7)	–2.7 (–10.7 to 5.3)	10.5 (0.9 to 20.1)	.03
DTR-QOL domain 2	69.6 (59.2 to 80.0)	5.1 (–3.8 to 14.0)	63.4 (53.5 to 73.4)	1.8 (–6.8 to 10.3)	3.3 (–6.9 to 13.5)	.51
DTR-QOL domain 3	81.1 (68.5 to 93.8)	–3.2 (–17.1 to 10.6)	80.7 (68.5 to 92.8)	–8.2 (–21.5 to 5.1)	5.0 (–10.9 to 20.9)	.53
DTR-QOL domain 4	42.1 (34.1 to 50.1)	–5.2 (–14.7 to 4.4)	43.2 (35.5 to 50.8)	–8.2 (–17.3 to 1.0)	3.0 (–7.9 to 14.0)	.58
DTR-QOL total	66.7 (58.4 to 74.9)	3.8 (–2.5 to 10.0)	61.9 (54.0 to 69.8)	–3.0 (–9.0 to 3.0)	6.7 (–0.5 to 13.9)	.07

a PAID, Problem Areas in Diabetes Survey.

b DTR-QOL, Diabetes Therapy-Related QOL.

Regarding patient satisfaction, improvements in PAID (−7.6, 95% CI −13.7 to −1.4; *P*=.02) and DTR-QOL domain 1 scores (10.5, 95% CI 0.9‐20.1; *P*=.03) from the baseline were significantly greater in the telemedicine group than in the standard care group. Changes in DTR-QOL domain 2, 3, and 4 scores were comparable between the 2 groups.

### Secondary Outcomes

Maternal metabolic parameters and perinatal outcomes are shown in Tables4  5. We observed a trend toward greater maternal weight gain in the telemedicine group; however, the difference was not statistically significant (group difference in change: 1.1 kg, 95% CI −0.6 to 2.7 kg; *P*=.19). Furthermore, the SBP and DBP decreased in the telemedicine group and increased in the control group; however, these differences were not significant (change in SBP between the groups: −8.4 mm Hg, 95% CI −17.7 to 0.9 mm Hg; *P*=.08; DBP difference: −5.5 mm Hg, 95% CI −13.9 to 3.0 mm Hg; *P*=.20). Changes in glucose and lipid parameters were similar between the groups.

**Table 4.** Secondary maternal outcomes.

Variables	Telemedicine group (n=18)		Standard care group (n=20)		Group comparison	
	After intervention (95% CI)	Change from baseline (95% CI)	After intervention (95% CI)	Change from baseline (95% CI)	Difference in change (95% CI)	*P* value
Maternal outcomes						
Maternal body weight (kg)	67.6 (62.7 to 72.4)	3.8 (2.4 to 5.2)	61.5 (56.9 to 66.2)	2.7 (1.4 to 4.1)	1.1 (−0.6 to 2.7)	.19
BMI (kg/m^2^)	26.2 (24.6 to 27.7)	1.5 (0.9 to 2.0)	24.6 (23.1 to 26.0)	1.0 (0.5 to 1.6)	0.4 (−0.2 to 1.0)	.22
Systolic blood pressure (mm Hg)	108.5 (103.0 to 113.9)	−2.8 (−10.9 to 5.3)	116.3 (111.1 to 121.5)	5.6 (−2.2 to 13.3)	−8.4 (−17.7 to 0.9)	.08
Diastolic blood pressure (mm Hg)	66.9 (61.4 to 72.4)	−0.5 (−7.8 to 6.9)	70.4 (65.1 to 75.7)	5.0 (−2.1 to 12.1)	−5.5 (−13.9 to 3.0)	.20
Plasma glucose (mg/dL)	101.1 (86.5 to 115.7)	−2.3 (−19.8 to 15.2)	93.4 (79.4 to 107.4)	1.6 (−15.1 to 18.4)	−4.0 (−24.0 to 16.1)	.69
HbA_1c_^a^ (%)	5.4 (5.3 to 5.6)	0.1 (−0.1 to 0.2)	5.4 (5.3 to 5.6)	0.2 (0.0 to 0.3)	−0.1 (−0.3 to 0.1)	.33
HbA_1c_ (mmol/mol)	35.7 (33.9 to 37.5)	0.8 (−0.9 to 2.5)	35.9 (34.1 to 37.6)	1.8 (0.2 to 3.4)	−0.9 (−2.9 to 1.0)	.33
GA^b^ (%)	11.8 (11.2 to 12.4)	−0.3 (−0.7 to 0.1)	12.3 (11.7 to 12.8)	−0.3 (−0.7 to 0.1)	0.0 (−0.5 to 0.4)	.92
C peptide (ng/mL)	5.4 (2.8 to 8.0)	0.6 (−2.0 to 3.3)	2.6 (0.1 to 5.1)	0.1 (−2.4 to 2.7)	0.5 (−2.6 to 3.5)	.75
LDL-C^c^ (mg/dL)	140.6 (119.0 to 162.2)	22.3 (8.5 to 36.1)	148.2 (127.5 to 168.9)	25.3 (12.0 to 38.5)	−3.0 (−18.8 to 12.9)	.71
HDL-C^d^ (mg/dL)	76.3 (67.1 to 85.5)	3.2 (−2.1 to 8.6)	77.1 (68.4 to 85.9)	1.5 (−3.6 to 6.7)	1.7 (−4.5 to 7.9)	.58
Triglyceride (mg/dL)	298.6 (227.6 to 369.7)	56.0 (−1.3 to 113.3)	233.3 (165.1 to 301.4)	44.3 (−10.6 to 99.3)	11.6 (−54.2 to 77.4)	.72
Urinary albumin-to-creatinine ratio (mg/gCr)	10.9 (−1.2 to 23.0)	1.0 (−10.2 to 12.2)	14.4 (2.8 to 26.0)	7.5 (−3.2 to 18.2)	−6.5 (−19.4 to 6.3)	.31
Total insulin dose (U/day)	18.0 (11.2 to 24.8)	9.3 (4.2 to 14.4)	16.9 (10.4 to 23.4)	9.3 (4.4, to 14.2)	0.0 (−5.8 to 5.9)	.99
Proportion of target glucose levels in the previous 7 days (%)	78.8 (70.7 to 86.9)	5.9 (−2.9 to 14.7)	84.6 (76.2 to 92.9)	−2.5 (−11.6 to 6.6)	8.4 (−1.7 to 18.4)	.10
Outpatient consultation times (s)	543.7 (490.2 to 597.3)		518.2 (466.8 to 569.6)		—^e^	.41

a HbA_1c_: hemoglobin A_1c_.

b GA: glycated albumin.

c LDL-C: low-density lipoprotein cholesterol.

d HDL-C: high-density lipoprotein cholesterol.

e Not applicable.

**Table 5.** Secondary perinatal outcomes.

Variables	Telemedicine group (n=18)	Standard care group (n=20)	*P* value
Perinatal outcomes			
Gestational age at delivery (wk), median (IQR)	39 (38-39)	38 (38-39)	.43
Mode of delivery, n (%)			
Vaginal delivery	12 (67)	9 (45)	.18
Cesarean section	6 (33)	11 (55)	—^a^
Cesarean section, n (%)			
Planned	2 (33)	4 (36)	.90
Unplanned	4 (67)	7 (64)	—
Premature rupture of the membrane, n (%)	2 (11)	2 (10)	.91
Postpartum hemorrhage, n (%)	0 (0)	0 (0)	—
Shoulder dystocia, n (%)	0 (0)	0 (0)	—
Gestational hypertension, n (%)	1 (6)	0 (0)	.29
Infant sex, n (%)			
Male	10 (56)	8 (40)	.34
Female	8 (44)	12 (60)	—
Birth length (cm) (95% CI)	47.5 (45.8 to 49.2)	47.0 (45.4 to 48.7)	.67
Birth weight (g) (95% CI)	2969 (2679 to 3260)	2826 (2548 to 3105)	.39
Neonatal hypoglycemia, n (%)	7 (39)	8 (40)	.94
Apgar score 1 min (95% CI)	8.1 (7.5 to 8.7)	8.0 (7.5 to 8.6)	.79
Apgar score 5 min (95% CI)	8.9 (8.4 to 9.4)	8.8 (8.4 to 9.3)	.76
Umbilical artery pH (95% CI)	7.297 (7.265 to 7.329)	7.310 (7.280 to 7.341)	.46
Macrosomia, n (%)	0 (0)	0 (0)	—
Low birth weight infant, n (%)	3 (17)	3 (15)	.89
Large for gestational age, n (%)	2 (11)	3 (15)	.72
Small for gestational age, n (%)	1 (6)	3 (15)	.34
Congenital diseases, n (%)	1 (6)	0 (0)	.29
Admission to NICU^b^, n (%)	1 (6)	2 (10)	.61
Outpatient consultation times (s) (95% CI)	543.7 (490.2 to 597.3)	518.2 (466.8 to 569.6)	.41

a Not applicable.

b NICU: neonatal intensive care unit.

Gestational age at delivery, mode of delivery, and the frequency of other perinatal complications were similar between the groups. There were no significant differences in neonatal height, weight, frequency of neonatal hypoglycemia, Apgar score, umbilical artery pH, or admission to the neonatal intensive care unit. Additionally, the time required for consultations per person was similar between the groups (543.7, 95% CI 490.2‐597.3 s vs 518.2 95% CI 466.8‐569.6 s, *P*=.41).

## Discussion

### Principal Findings

This study demonstrates that telemedicine use for GDM management improves patient satisfaction and reduces total costs, with comparable glycemic control and perinatal complications between the 2 groups. Thus, mHealth-supported telemedicine in the management of GDM can be a cost-saving technology providing better patient satisfaction. To the best of our knowledge, this is the first study to evaluate the impact of telemedicine on both psychological burden and total costs in patients with GDM.

### Comparison With Previous Studies

Several previous studies have examined the effects of interventions through telemedicine or mobile apps for patients with GDM, in addition to regular consultations. These studies reported a reduced incidence of metabolic syndrome [24], decreased weight gain [25], higher rates of achieving target blood glucose levels [26], and a shorter time to reach target blood glucose levels [27]. Our findings share similarities with previous studies reporting a slight improvement in satisfaction scores with a mobile phone–based glucose management system [12] and a reduction in direct medical costs [13]. However, important differences remain because those earlier studies were performed alongside regular in-person consultations and did not evaluate the direct non–health care costs and indirect costs.

### Clinical and Economic Implications

The impact of telemedicine on costs for patients with GDM has been previously reported [12,13]; however, these reports focused solely on direct health care costs. In our study, we evaluated the direct non–health care costs and indirect costs, accounting for a significant portion of the actual patient and societal burdens. The evaluation revealed that both costs were significantly lower in the telemedicine group, which may have contributed to the improvement in patient satisfaction. Although findings from a systematic review suggest that telemedicine holds a favorable level of economic efficiency [28], reports on the effectiveness and safety of telemedicine for managing GDM in Japan are lacking. Japan’s health care system uses a universal coverage model, and medical services are priced using a unified fee schedule. The typical copayment rate is 30%, with the remainder covered by public health insurance, ensuring universal access to health care. Moreover, the official fees for telemedicine are lower than those for face-to-face consultations in Japan, which is presumed to be a factor hindering its broader adoption. Considering the positive impact on the economic and psychological burdens shown in this study, we believe that these findings could support raising the official fee for telemedicine to the same level as regular consultations for patients with GDM.

Regarding the safety of telemedicine as a replacement for regular clinical visits, an RCT comprising 100 patients with GDM reported that HbA_1c_ levels, the rate of cesarean sections, and the rate of LGA infants were comparable between the groups [29]. Furthermore, a similar report in 2023 indicated that there was no deterioration in glycemic control or increase in perinatal complications and that the telemedicine group had a significantly higher rate of achieving postprandial blood glucose targets [30]. Considering these previous research results, the safety of replacing regular clinical visits with telemedicine has been demonstrated. Most meta-analyses examining the effectiveness of telemedicine in patients with GDM combined studies that used telemedicine in addition to regular clinical visits with those that used telemedicine as a replacement. These analyses also consistently showed favorable results for telemedicine, including improvements in HbA_1c_ levels [10,31] and reductions in the risk of perinatal complications [11]. In our study, the 2 groups exhibited comparable glycemic control and perinatal outcomes, reaffirming the safety as reported in previous studies. The improvements in glycemic control and a reduction in perinatal complications, as observed in other studies, were not seen in this study possibly because of the sample size of this study. Additionally, it was speculated that the interventions of telemedicine or applications, which were conducted in other studies alongside routine care, may have influenced the results.

In this study, the consultation time was measured as an exploratory evaluation item. Considering the preparation required for the telemedicine system, we anticipated that the consultation time would be longer in the intervention group. However, the actual consultation time was comparable between the groups, suggesting that the burden on health care providers was similar in both groups, thereby highlighting the value of the reduced costs and psychological burden observed in the telemedicine group.

### Policy Implications

The findings of this study have important implications for health policy and clinical practice. The observed reductions in psychological burden and costs suggest that structured telemedicine pathways may serve as an efficient alternative to conventional in-person GDM management. Incorporating such pathways into routine prenatal diabetes care has the potential to reduce patient time burdens and improve access to care. To achieve broader implementation, it will be essential to establish reimbursement models that appropriately cover telemedicine-based follow-up visits and digital monitoring.

### Limitations

This study has some limitations. First, due to its design, blinding was challenging, which may have introduced clinical bias. Second, the study primarily involved Japanese patients residing in urban areas; hence, further research is needed in other settings and among different ethnic groups. Third, the small sample size reduced the power to detect infrequent complications evaluated as secondary outcomes. Fourth, we did not consider internet communication costs or the expenses associated with implementing the telemedicine system in medical institutions. While it is assumed that most participants conducted their consultations in an environment with Wi-Fi, additional costs may have been incurred. Finally, the questionnaire used was originally designed for patients with diabetes mellitus, not GDM. This limitation was communicated to participants on a separate sheet as a precaution.

### Conclusions

In conclusion, TELEGLAM is the first study to examine the impact of telemedicine on psychological burdens and total costs in patients with GDM. In this study, we demonstrated that substituting regular consultations with telemedicine for patients with GDM reduced both psychological and economic burdens. Our results underscore the potential of mHealth-enabled telemedicine as a safe and effective substitute for conventional in-person GDM management, and we anticipate that these findings will support its broader implementation in clinical care. To translate these benefits into practice, health systems should integrate telemedicine into routine prenatal diabetes care and adopt policies that reduce financial and psychological burdens for patients.

## Supplementary material

10.2196/72242 Multimedia Appendix 1 Health care costs in the case where telemedicine costs were equivalent to those of face-to-face consultations.

10.2196/72242 Checklist 1 CONSORT-EHEALTH checklist.

## Abbreviations

CCA: cost-consequence analysis
DBP: diastolic blood pressure
DTR-QOL: Diabetes Therapy-Related QOL
GDM: gestational diabetes mellitus
HbA_1c_: hemoglobin A_1c_
LGA: large-for-gestational-age
mHealth: mobile health
OGTT: oral glucose tolerance test
PAID: the Problem Areas in Diabetes Survey
PPP: purchasing power parity
QOL: quality of life
RCT: randomized controlled trial
SBP: systolic blood pressure
