# Supplementary material for: Efficacy and Safety of a Telemedicine System in Patients With Gestational Diabetes Mellitus (TELEGLAM): Single-Center, 2-Arm, Randomized, Open-Label, Parallel-Group Study
Source: JMIR Mhealth Uhealth. 2026 May 8;14:e72242. doi: 10.2196/72242 (PMC13155504; doi:10.2196/72242)
Supplement: Multimedia Appendix 1 [file mhealth-v14-e72242-s001.docx]

Supplementary Table 1. Healthcare costs in the case where telemedicine costs were equivalent to those of face-to-face consultations.

| Variables | Telemedicine group (n=18) | | Standard care group (n=20) | | Mean difference | | *P* value |
| --- | --- | --- | --- | --- | --- | --- | --- |
| Costs | yen | PPP-adjusted  dollars | yen | PPP-adjusted  dollars | yen | PPP-adjusted  dollars |  |
| Direct healthcare costs | 23026 (20295, 25757) | 200 (176, 224) | 23737 (21117, 26358) | 206 (184, 229) | -711 (-3849, 2427) | -6 (-33, 21) | .65 |
| Direct non-healthcare costs | 922 (-240, 2083) | 8 (-2, 18) | 2561 (1447, 3676) | 22 (13, 32) | -1639 (-2974, -304) | -14 (-26, -3) | .02 |
| Indirect costs | 8981 (-7119, 25082) | 78 (-62, 218) | 32832 (17384, 48279) | 285 (151, 420) | -23851 (-42349, -5353) | -207 (-368, -47) | .01 |
| Total costs | 32929 (15635, 50223) | 286 (136, 437) | 59130 (42538, 75722) | 514 (370, 658) | -26201 (-46070, -6332) | -228 (-401, -55) | .01 |

Data are presented as estimated values (95%CI), unless otherwise stated.

Abbreviations: PPP, purchasing power parity.
